# Enhancement of *Aedes aegypti* susceptibility to dengue by *Wolbachia* is not supported

**DOI:** 10.1038/s41467-020-19830-6

**Published:** 2020-11-30

**Authors:** Thomas H. Ant, Maria-Vittoria Mancini, Julien Martinez, Steven P. Sinkins

**Affiliations:** Centre for Virus Research, University of Glasgow, Glasgow, G12 8QQ UK

**Keywords:** Ecological epidemiology, Pathogens

**Arising from** King et al. *Nature Communications* 10.1038/s41467-018-03981-8 (2018).

Releases of *Aedes aegypti* mosquitoes carrying *Wolbachia* to block the transmission of dengue virus (DENV) are currently being deployed as a novel dengue control strategy in a number of countries, with very encouraging results^1,2^. In their paper entitled “Variation in *Wolbachia* effects on *Aedes* mosquitoes as a determinant of invasiveness and vectorial capacity”, King et al.^3^ used DENV infection and transmission modelling to reinterpret experimental data from two previous studies^4,5^. The authors claimed that *w*Mel *Wolbachia* increase the mean susceptibility of *Ae. aegypti* to DENV, contradicting various other studies^6–12^. Here, we raise concerns with the experimental approaches used to generate one of the primary datasets on which the modelling is based, and we discuss how these limitations could make some of the original conclusions misleading.

King et al.^3^ base a large part of their modelling analysis on experimental data reported in Souto-Maior et al.^4^, consisting of intrathoracic challenges with DENV-1 of *w*Mel-carrying and tetracycline-cured (TET) *Ae. aegypti*, with virus quantified by qRT-PCR in whole bodies. An equivalent proportion of dengue-positive *w*Mel-carrying mosquitoes compared to TET controls is reported at an injection titre of 10^4^ TCID50/ml, a slight increase in positives in *w*Mel-carrying mosquitoes at 10^5^ and 10^6^ TCID50/ml, and a slight decrease at 10^7^ and 10^8^ TCID50/ml. Intrathoracic inoculation can be a convenient first approximation of infectivity since it is easier to perform than oral challenge; however it bypasses the biologically crucial midgut infection barrier, a key site of virus blocking by *Wolbachia*^13^. Thus, it represents a substantial deviation from the natural infection route, and its biological relevance is especially questionable given the binary presence/absence of viral RNA used by King et al.^3^ as the measure of susceptibility.

Furthermore, in Souto-Maior et al.^4^ a high proportion of the mosquitoes assigned as DENV-infected, particularly those at low virus titre challenges, are likely false positives. Examination of the raw qPCR cycle threshold (Ct) values reveals a positive signal in 5 out of 35 negative control mosquitoes following mock challenge. The mean of these Ct values is lower, indicating a stronger signal, than many of the Ct values from mosquitoes counted as DENV-infected (Fig. 1a). A strong signal in the negative control leads to uncertainty over the specificity of the qPCR assay used and suggests that many of the readings indicating DENV-positivity from the 10^4^, 10^5^, 10^6^, and 10^7^ TCID50/ml inoculations could be artefacts. The very low infection rates at the lower virus inoculation titres are similar to the false positive rate of the negative controls: 4% (0.4–13.7%), 8.8% (1.8–23.7%), and 18.4% (8.4–30.9%), for 10^4^, 10^5^, and 10^6^ TCID50/ml, respectively, compared to 14.3% (4.8–30.3%) for negative controls, where all percentage values in parentheses are exact binomial 95% CI.

**Fig. 1:**
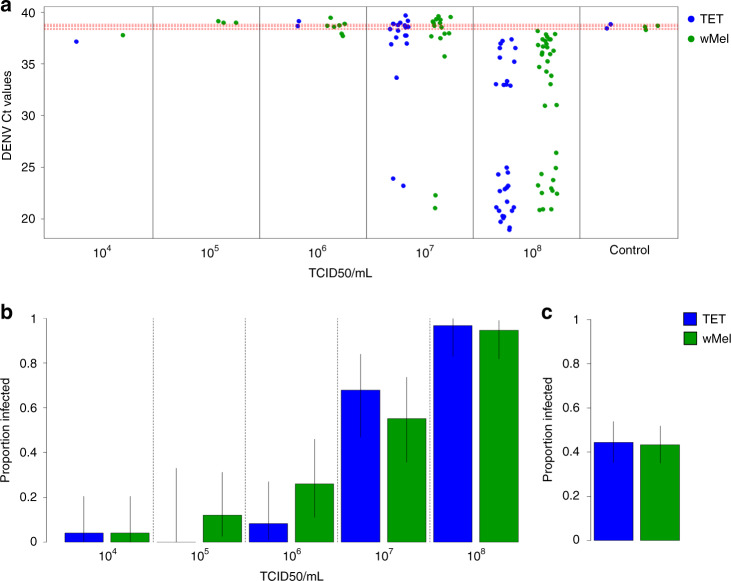
qPCR data and DENV infection rates from the original Souto-Maior et al.^4^ data. **a** Raw DENV qPCR Ct values resulting from intrathoracic inoculations. Blue and green dots show Ct values from TET and *w*Mel mosquitoes, respectively. Red dashed lines show Ct values from mock-injected negative controls. **b**–**c** Proportions of TET and *w*Mel mosquitoes classified by Souto-Maior et al.^4^ (and likewise by King et al.^3^) as qPCR positive—proportions have been recalculated from the original dataset. Error bars show 95% binomial confidence intervals.

Despite a low number of virus-positive mosquitoes at the lower inoculation titres, with a total of 14 positive mosquitoes across both the *w*Mel and TET groups for the 10^4^, 10^5^, and 10^6^ TCID50/ml inoculations combined, resulting in low statistical power (Fig. 1b), King et al.^3^ fit a dose-response model to the data and conclude that “*Wolbachia* increase mean susceptibility [to infection] by a factor of 6.9”. Even if all DENV-positive PCR readings are assumed to be valid, this increase in susceptibility is difficult to reconcile with the data. The total proportion of DENV-positive TET mosquitoes was 52 out of 117 or 44.4% (35.3–53.9%) compared to 62 out of 143 or 43.3% (35.1–51.9) for *w*Mel, a slight decrease in infection rate that was not statistically significant: *p* = 0.96, Binomial test (Fig. 1c). There are also very low infection rates and infection titres in the Souto-Maior et al.^4^ dataset even at relatively high inoculation titres, e.g., an 8.3% infection rate in TET mosquitoes following DENV-inoculation at 10^6^ TCID50/ml. This result is inconsistent with other studies: for example, 100% infection was observed following DENV inoculation at 10^4^ genome copies/ml^11^; 95% infection following DENV-inoculation at 10^5^ TCID50/ml^10^; and 100% infection following DENV inoculation at 6 × 10^5^ PFU/ml^14^. Finally, there are issues with data transfer, since King et al.^3^ present the *w*Mel cohort as having a higher proportion DENV infected at 10^4^ TCID50/ml, whereas in the Souto-Maior et al.^4^ dataset the proportions are equivalent.

The second dataset used by King et al.^3^ was from Ferguson et al.^5^, comparing *w*Mel-carrying and *Wolbachia*-negative *Ae. aegypti* fed on viremic blood from dengue patients (serotypes 1–4). The study provides data on DENV infection rates and titres in dissected abdomens by qRT-PCR, and a direct assessment of the infectivity of mosquito saliva. Applying their dose-response modelling to proportions of infected abdomens, King et al.^3^ conclude an “increase in mean [*w*Mel-infected] mosquito susceptibility to infection to a factor of 1.5” relative to *Wolbachia*-negative controls. However, the value is misleading. Replotting the Ferguson et al.^5^ abdomen infection rates (Fig. 2) shows that in 23 out of 42 feedings or 54.7% (38.67–70.2%), *w*Mel-carrying mosquitoes displayed a lower infection proportion than *Wolbachia*-negative controls; in 14 out of 42 or 33.3% (19.6–49.6%) the proportions were exactly equivalent; and in only 5 out of 42 or 11.9% of feedings (3.9–25.6%) was the proportion infected higher in *w*Mel-carrying mosquitoes. Thus 665 out of a total 955 or 69.6% (66.6–72.5%) of *Wolbachia-*negative mosquitoes developed abdominal infections, compared to 507 out of a total 877 or 57.8% (54.4–61.1%) of *w*Mel-carrying mosquitoes, a 16.9% decrease in infection rate: *p* = 0.0147, Fisher’s exact test.

**Fig. 2:**
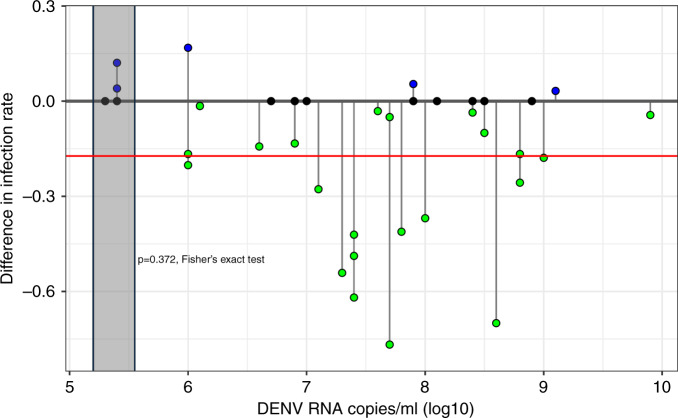
Differences in abdomen infection rate [(rate in *w*Mel)—(rate in *Wolbachia*-uninfected)] between *w*Mel and *Wolbachia*-negative mosquitoes fed on viremic blood, replotted from the original Ferguson et al.^5^ data. Each dot represents a feeding on blood sourced from a different dengue-infected patient. Negative differences (green dots) indicate a lower abdomen infection rate in *w*Mel-carrying mosquitoes. Positive differences (blue dots) indicate a higher abdomen infection rate in *w*Mel-carrying mosquitoes. Black dots indicate no difference. The red line shows the mean difference across all DENV titres. Shaded area shows data points in the 10^5^ ≤ X < 10^6^ RNA copies/ml stratum and statistical test.

In their susceptibility analysis, King et al.^3^ stratify the Ferguson et al.^5^ abdomen data into groups of Log10 viral titre i.e., patients with 10^5^ ≤ X < 10^6^ and 10^6^ ≤ X < 10^7^ RNA copies/ml etc. are grouped independently. Using this stratification system King et al. observe that there is an increased infection rate in the *w*Mel cohort in the 10^5^ ≤ X < 10^6^ grouping. However, while 14 out of 75 or 18.7% (10.6–29.3%) of *w*Mel carriers were DENV positive in abdomens compared to 9 out of 80 or 11.3% (5.3–20.3%) of *Wolbachia* negatives, this difference is not statistically significant: *p* = 0.372, Fisher’s exact test (Fig. 2). At all other strata the proportion of infected mosquitoes was lower in the *w*Mel cohort. There was also no consideration of differences in output viral titres in challenged mosquitoes in the King et al.^3^ model; while in Ferguson et al.^5^ the 10^5^ ≤ X < 10^6^ stratum shows a slight increase in infection rate for *w*Mel, virus titre in the same mosquitoes is on average 10–100-fold less. The need for consideration of virus in the saliva is also paramount.

Evaluations of safety and efficacy of the *Wolbachia* replacement approach are valuable, but must be based on robust experimental foundations and analysis, given that misleading ‘headline’ statements can adversely impact stakeholder perceptions. Given low-titre asymptomatic carriers of DENV may be important contributors to its transmission^15^, the study by King et al.^3^ could even be interpreted as providing evidence of a risk that *Wolbachia* deployment could actually increase dengue incidence. *Wolbachia* replacement requires the release of biting female mosquitoes, and high levels of community support are thus essential; the intervention is particularly sensitive to any safety concerns. The limitations in the experimental dataset on which King et al.^3^ based their analysis suggest that caution is needed when interpreting their argument for enhanced mean DENV susceptibility in *Ae. aegypti* carrying *w*Mel *Wolbachia*.

## Reporting summary

Further information on research design is available in the Nature Research Reporting Summary linked to this article.

## Supplementary information

Reporting Summary

## Data Availability

There is no new data associated with this manuscript.
